# RELATIONSHIP BETWEEN INFORMAL AND FORMAL HELP-SEEKING BEHAVIORS FOR ELDER MISTREATMENT AMONG CHINESE IMMIGRANTS

**DOI:** 10.1093/geroni/igad104.2472

**Published:** 2023-12-21

**Authors:** Ying-Yu Chao, Jin Young Seo, Yu-Ping Chang

**Affiliations:** Rutgers, the State University of New Jersey, Somerset, New Jersey, United States; Hunter College, CUNY, New York City, New York, United States; University at Buffalo, The State University of New York, Buffalo, New York, United States

## Abstract

**Purpose:**

Help-seeking minimizes the consequences from elder mistreatment (EM). However, formal help-seeking could be challenging for older immigrants due to cultural and linguistic barriers. Informal supporters have been shown to promote formal help-seeking among EM victims. This study aimed to examine whether informal help facilitates U.S. Chinese EM victims with seeking help from formal sources.

**Methods:**

Data were derived from the Population Study of Chinese Elderly in Chicago. EM measurements included psychological, physical, sexual mistreatment, financial exploitation, and caregiver neglect. Seeking help from informal and formal sources were evaluated. Descriptive statistics and multinominal logistic regression were conducted.

**Results:**

Among 450 EM victims, 53.48% of them sought informal help, 31.75% did not seek any help, 11.42% sought both informal and formal help, and 3.34% sought formal help. The most common informal sources were adult children, partners, and friends/neighbors. The most common formal sources were community social services organizations, followed by legal criminal justice systems. Compared to those who did not seek any help, victims who reported more emotional closeness within their social network and experienced financial exploitation were more likely to seek help from formal sources. However, informal help-seeking was not significantly associated with formal help-seeking. Conclusion/implication: The findings suggest that most U.S. Chinese older adults who experienced EM did not seek formal help. Higher levels of contact with social networks could increase seeking help from formal sources. However, future research is needed to explore why informal supporters did not facilitate seeking help from formal sources in Chinese EM victims.

